# Spatio-temporal variation in prostate cancer testing in Stockholm: A population-based study

**DOI:** 10.1371/journal.pone.0308254

**Published:** 2024-08-15

**Authors:** Balram Rai, Tobias Nordström, Anna Lantz, Rolf Lyneborg Lund, Ralf Kuja-Halkola, Marta Rado, Sara Öberg, Shuang Hao, Xiaoyang Du, Mark Clements

**Affiliations:** 1 Department of Medical Epidemiology and Biostatistics, Karolinska Institutet, Stockholm, Sweden; 2 Department of Sociology and Social Work, Aalborg University, Aalborg Øst, Denmark; Jan Biziel University Hospital No 2 in Bydgoszcz: Szpital Uniwersytecki Nr 2 im dr Jana Biziela w Bydgoszczy, POLAND

## Abstract

Prostate cancer screening using prostate-specific antigen (PSA) testing is controversial but remains prevalent in many countries. There is little information in Sweden or elsewhere on the spatial variation in PSA testing. This study aims to describe the spatio-temporal variation in PSA testing prior to a prostate cancer diagnosis in the Stockholm region at the municipality and small area levels. A population-based register study comprised men aged 40 years and over living in the Stockholm region during 2007–2016. For Stockholm in 2016, we reported the proportion of men who had a PSA test for the preceding one, two, five and ten years by ten-year age groups. The age-standardised proportion of men having a PSA test was reported for municipalities by calendar years. We used spatial smoothing for calculating the age-standardised proportion of men having a PSA test in a small area for each calendar year. In 2016, 74.0% and 77.8% of men aged 60–69 and 70–79 years respectively had taken a PSA test in the previous ten years. The municipalities of Danderyd and Ekerö showed high proportions of PSA testing. A marked heterogeneity in such proportions within each municipality was observed. The odds ratio for having a PSA test for those born in Sweden was 2.22 (95% CI 2.00–2.52). Opportunistic PSA testing is widespread with three quarters of men in their sixties and seventies having had a test in the preceding decade. We found evidence for marked geographical heterogeneity, where more affluent and metropolitan areas had higher levels of testing. Variations in PSA testing was associated with socio-economic position and demographic factors including education, income and country of birth.

## Introduction

Prostate cancer is the most common types of cancer among men globally. The incidence of prostate cancer has been increasing in Sweden with a gradual decline in mortality rates [1]. The increasing incidence can be partially attributed to the introduction of prostate-specific antigen (PSA) testing to the general population [2]. PSA is a glycoprotein produced by the prostate gland, which might be elevated due to several reasons, including cancer, benign prostatic hyperplasia and subclinical prostatic inflammation [3, 4]. The epidemiology of prostate cancer remains complex and there are few opportunities for primary prevention of the disease [5]. Screening using the PSA test remains controversial, with evidence that screening with PSA testing may reduce the prostate cancer mortality but at the cost of overdiagnosis and overtreatment [6, 7]. It is unclear whether the benefits of early detection outweigh the potential short- and long-term harms due to prostate cancer diagnostics and treatment [8, 9]. Nevertheless, the wide availability and affordability of the PSA test has resulted in high levels of opportunistic screening in many countries.

Many countries have recommended against population-based PSA screening for prostate cancer due to its potential harms [10]. In 2022, the European Council recommended that member states use a stepwise approach to organised prostate cancer testing using PSA testing in combination with magnetic resonance imaging (MRI) [11]. A literature review [12] found evidence for geographic differences in PSA testing, where metropolitan and affluent areas tended to have had higher levels of testing. There has been broad uptake of PSA testing with variations among socio-economic groups within and across many European countries [13]. Using data for Stockholm through to 2011, the use of PSA tests was prevalent in Stockholm [14]. There is also evidence for high levels of PSA testing in other parts of Sweden [15]. Several recent pilot studies are assessing the feasibility of organised prostate cancer testing in Sweden [16, 17]. Nordström and colleagues [18] described socio-economic gradients in PSA testing for education in Stockholm and found that PSA testing was more common for those with longer education. However, there is little information in Sweden or elsewhere on spatial heterogeniety at the small area level in PSA testing. The association of PSA testing with other measures of socio-economic position (SEP), such as income and country of birth, in Stockholm have not been previously described.

The aim of this study is to depict the variation in PSA testing patterns among men living in Stockholm by age-groups, geographical areas, calendar years and test the association of PSA testing with education, income and country of birth. Specifically, we mapped the proportion of men being tested by municipalities and small areas over time, and investigate whether there is evidence for clustering of high or low levels of testing. We hypothesised that PSA testing will be more common in more affluent areas, and that there will be substantial clustering of PSA testing in those areas.

## Material and methods

### Data sources

All the information on PSA tests was obtained from the Stockholm PSA and Biopsy Register which includes data from the three laboratories (Karolinska University Laboratory, Aleris, and Unilabs) who analyse PSA tests performed in the Stockholm region. These data were linked with the population registers through Sweden’s unique identification number. The population in this study was men aged 40 years and over living in the Stockholm region during 2007–2016 with complete information on their small area of residence. For further details on the Stockholm PSA and Biopsy Register, see [19]. Age was restricted because PSA testing before age 40 years was very uncommon. For each year that a man resided in Stockholm, we had information on the man’s age, his small area of residence, whether he had had a PSA test and a prostate cancer diagnosis. Geographical geometry files for Stockholm and data for socio-economic position (SEP) at the municipality level and at the small area level were extracted from the geodata extraction tool [20] in Swedenwhich includes geodata from Lantmäteriet and Statistics Sweden (SCB).

This analysis was performed under the ethical approval (Dnr 2022-02464-02) for small area analysis of PSA testing from the Swedish Ethical Review Authority. The need for informed consent was waived as it was a register-based study. The data were accessed on 1^st^ August, 2022 for the research purposes. The data were analysed anonymously and we did not have access to the information that could potentially identify any individuals.

### SAMS small areas and socio-economic position

SAMS (Small Area Market Statistics) is a regional classification system by Statistics Sweden that delineates very small and socio-economically homogeneous residential areas. There were a total of 9206 SAMS areas in Sweden, with 890 areas in the Stockholm region. SAMS has been used to study health by small areas, including the effects of neighbourhood on health outcomes [21, 22]. We also used the data for SEP including education, income, and country of birth at the small area level. The SEP data were extracted for the year 2016 and used to model the association with PSA data for the same year. Education was categorised in terms of length of education as short (< = 9 years), intermediate (>9 and < = 12 years), and long (> 12 years). The categorisation of income was based on the disposable income brackets per annum by Statistics Sweden as low (< = 167,400 Swedish kronor), middle (167,401–333,192), and high (> 333,192). Country of birth was divided into two categories for men born in Sweden and born elsewhere.

### Statistical analysis

First we calculated the proportion of men without a prior prostate cancer diagnosis having a PSA test by ten-year age groups (40–49, 50–59, 60–69, 70–79, 80+) and calendar years from 2007 to 2016. For 2016, we reported the proportion of men who had a PSA test for the preceding one, two, five and ten years by ten-year age groups to see the long-term testing pattern. The age-standardised proportions of men having a PSA test were calculated for the municipalities by each calendar year. We used a spatial smoothing model for calculating the age-standardised proportion of men having a PSA test in a small area for each calendar year. The area-level association of PSA testing with the measures of SEP at the small area level in the year 2016 was reported in terms of odds ratios with 95% confidence interval (CI). The association of PSA testing with different measures were tested seperately to avoid the overlapping of the SEP measures between the areas. All the analyses were conditioned on men not having a prostate cancer diagnosis at the end of calendar year. The analyses focused on the number of men having a PSA test rather than the number of tests. All the statistical analyses were performed using R software version 4.2.2.

#### Spatial smoothing model

We used a Bayesian spatial model to predict the smoothed proportions of men having a PSA test in a small area for each calendar year. Let *Y*_*ij*_ represents the observed number of men taking PSA test in an area *i*, and age-group *j*, then *Y*_*ij*_ follows a binomial distribution, denoted *Bin*(*n*_*ij*_, *P*_*ij*_), where *n*_*ij*_ is the population of men in an area *i* and age-group *j* and *P*_*ij*_ is the probability for each man in the same population to have a PSA test

$$Y_{ij}=Bin\left(n_{ij},\;P_{ij}\right)$$

$$logit\left(P_{ij}\right)=\;\alpha \;+\;u_{i}\;+\;v_{i}+\;\beta_{j}\left(\text{age}\;\text{group}=j\right)$$

where *α* represents the intercept, *u*_*i*_ + *v*_*i*_ is an area random effect and *β*_*j*_ represents the coefficient for age-group *j*. The model allows each of the area to have its own intercept *α* + *u*_*i*_ + *v*_*i*_. The *u*_*i*_ are modelled using a conditional auto-regressive (CAR) prior distribution and *v*_*i*_ as independently and identically distributed normal variables. The spatial neighbourhood matrix for the CAR distribution was constructed using the notion of a queen contiguity matrix [23], where each area was informed by all areas with which it shared any border. The model was adjusted for age using a factor with ten year age groups. We used the R-INLA package [24] for fitting the model. After fitting the model, we obtained the predicted posterior probabilities for men taking a PSA test in an area each year. The proportions were standardised using Sweden´s national population in year 2000.

#### Spatial analysis

For the distribution of the proportion men having a PSA testing over time, we created four categories for proportion as <15%, 15-<20%, 20-<25%, and ≥25%. Furthermore, we used the local indicators of spatial association (LISAs) to identify the similarities and dissimilarities between small areas [25]. The LISA maps for the smoothed proportions depicted the clustering pattern among the SAMS areas in terms of high-high, low-low, high-low, and low-high clusters [26]. A high-high cluster indicated that a selected area had a high proportion and was also surrounded by areas (neighbours) with high proportions. The high-high and low-low clusters indicated a positive spatial autocorrelation and identified areas which were part of statistically significant clustering. We used the open-source GeoDa software for the spatial analysis.

## Results

**Fig 1:**
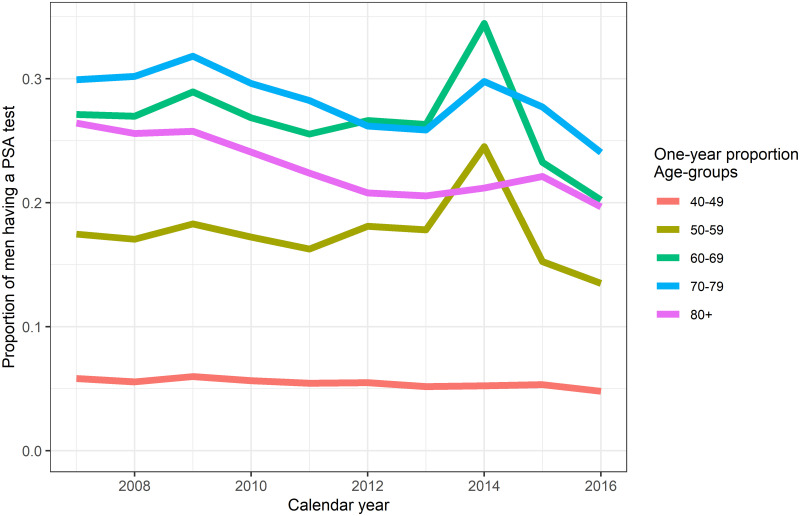
Proportion of men having a PSA test by age-group and calendar year in the Stockholm region, 2007–2016.

For Stockholm during 2007–2016, the PSA testing continued to be prevalent among older age-groups through to 2016 (Fig 1). The highest proportion of men having a PSA test belonged to those aged 70–79 years. The spike in the proportion of men testing for those aged 50–59 and 60–69 years in the year 2014 can be attributed to the population-based STHLM3 diagnostic study [27]. For men aged 70–79 years in 2016, 28.0% had a PSA test in the preceding year, while 77.8% had a PSA test in the preceding 10 years (Table 1). Over three-quarters of men aged 60 years and over had a PSA test in the preceding ten years. A prostate cancer diagnosis was more common in older ages, so there were higher proportions of men in older ages with a prior cancer diagnosis having a PSA test in last ten years (Table 1 in S1 Text). The odds ratio for men getting a PSA test if we assume a small area with all men born in Sweden was 2.22 (95% CI 2.00–2.52) compared to small area with all men born outside Sweden (Table 2). Small area with all men in high income category had a higher chance of getting a PSA test (OR 1.99, 95% CI 1.68–2.36) compared to small area with all men in low income category. Education was also significantly associated with PSA testing in the small area, where small area with all men having long educational length had a greater chance of a PSA test (OR 1.15, 95% CI 1.00–1.31).

**Table 1:** Prevalence of men having a PSA test in the Stockholm region by age group and 1, 2, 5, and 10 year prevalence, 2016.

Age group	Population	Prevalence of PSA testing in 2016 (%)			
		1 year	2 years	5 years	10 years
40–49	164160	5.2	8.9	15.0	20.7
50–59	141629	14.7	23.7	44.7	54.1
60–69	106826	22.6	34.3	62.3	74.0
70–79	77480	28.0	40.8	62.3	77.8
80+	32351	23.8	36.0	54.2	75.0

**Table 2:** Odds ratios for men having a PSA test by education, income and immigration in the Stockholm region in 2016.

Socio-economic Position	Odds ratio	95% CI
Education		
Short	Ref.	
Intermediate	1.62	(0.89, 2.95)
Long	1.15	(1.00, 1.33)
Income		
Low	Ref.	
Middle	1.41	(1.06, 1.87)
High	1.99	(1.68, 2.36)
Immigration		
Born elsewhere	Ref.	
Born in Sweden	2.22	(2.00, 2.52)

Ref.: Reference.

**Fig 2:**
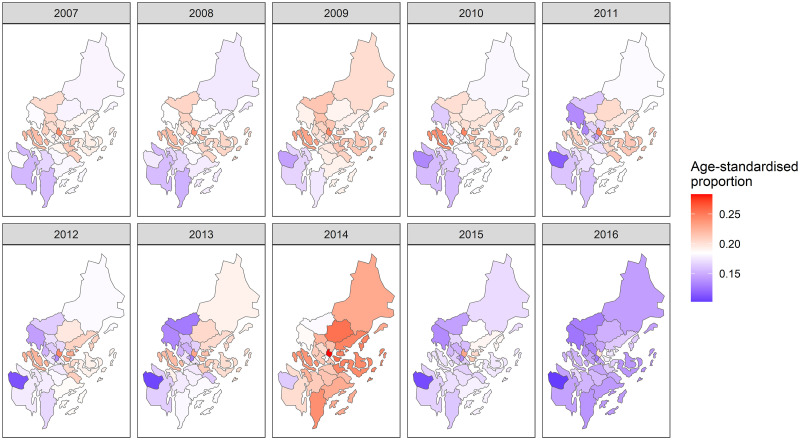
Proportion of men having a PSA test by municipalities and calendar year level in the Stockholm region, 2007–2016. PSA: prostate-specific antigen.

There was evidence for marked variation in PSA testing over time and between municipalities (Fig 2). The municipality with consistently the highest proportions of men having PSA test was the Danderyd municipality. After Danderyd, Ekerö had the second highest proportion of men taking a PSA test each year. The Nykvarn municipality had the lowest proportion of men having a PSA test for all except first two years. The municipalities in the central and eastern areas showed higher PSA test proportions compared to municipalities in the southern area. Some municipalities in the north-western area, including Sigtuna, Upplands Väsby and Upplands bro, had proportions that were initially high proportion and then decreased over time. The proportion of PSA testing decreased in all the municipalities towards the end of the study period.

**Fig 3:**
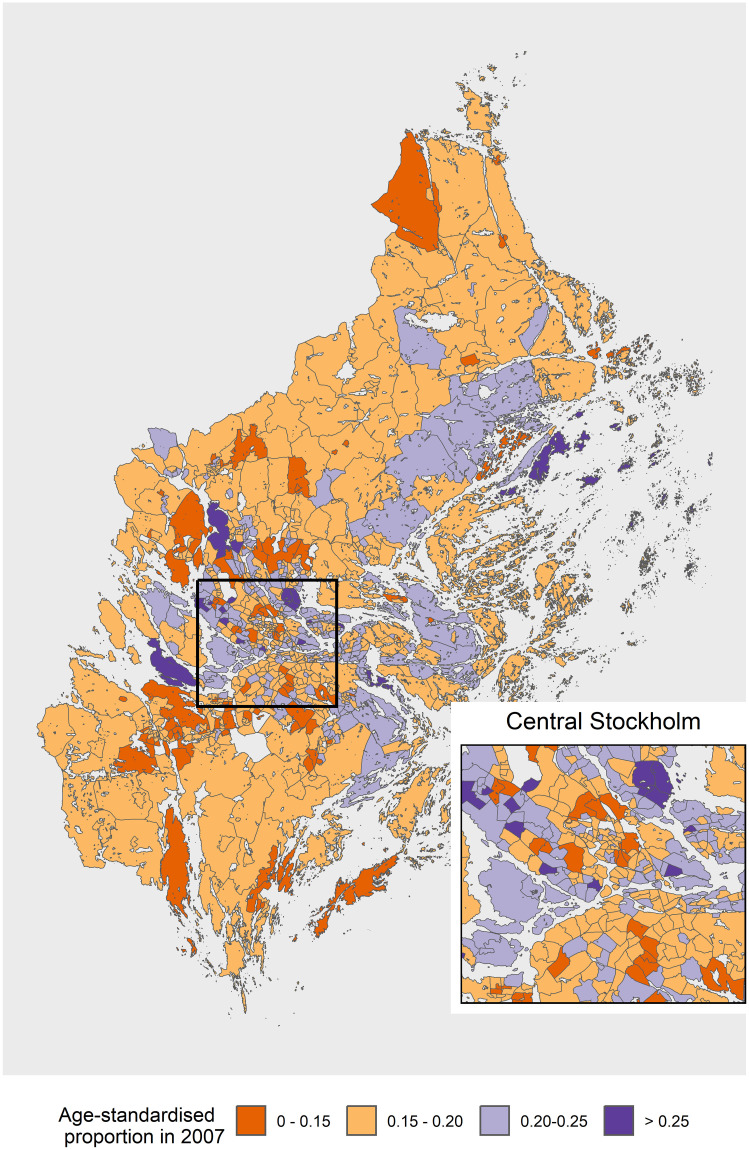
Proportion of men having a PSA test in Stockholm at small area level for year 2007. PSA: prostate-specific antigen; SAMS: Small Area Marketing Statistics.

**Fig 4:**
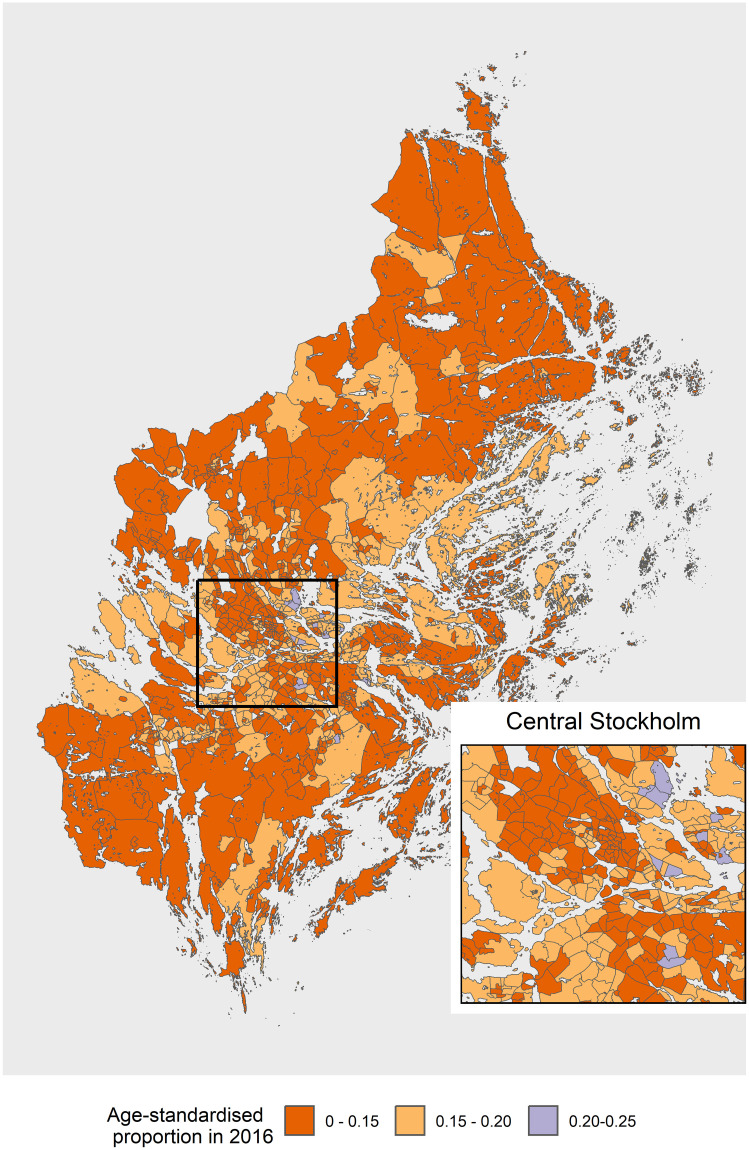
Proportion of men having a PSA test in Stockholm at small area level for year 2016. PSA: prostate-specific antigen; SAMS: Small Area Marketing Statistics.

**Fig 5:**
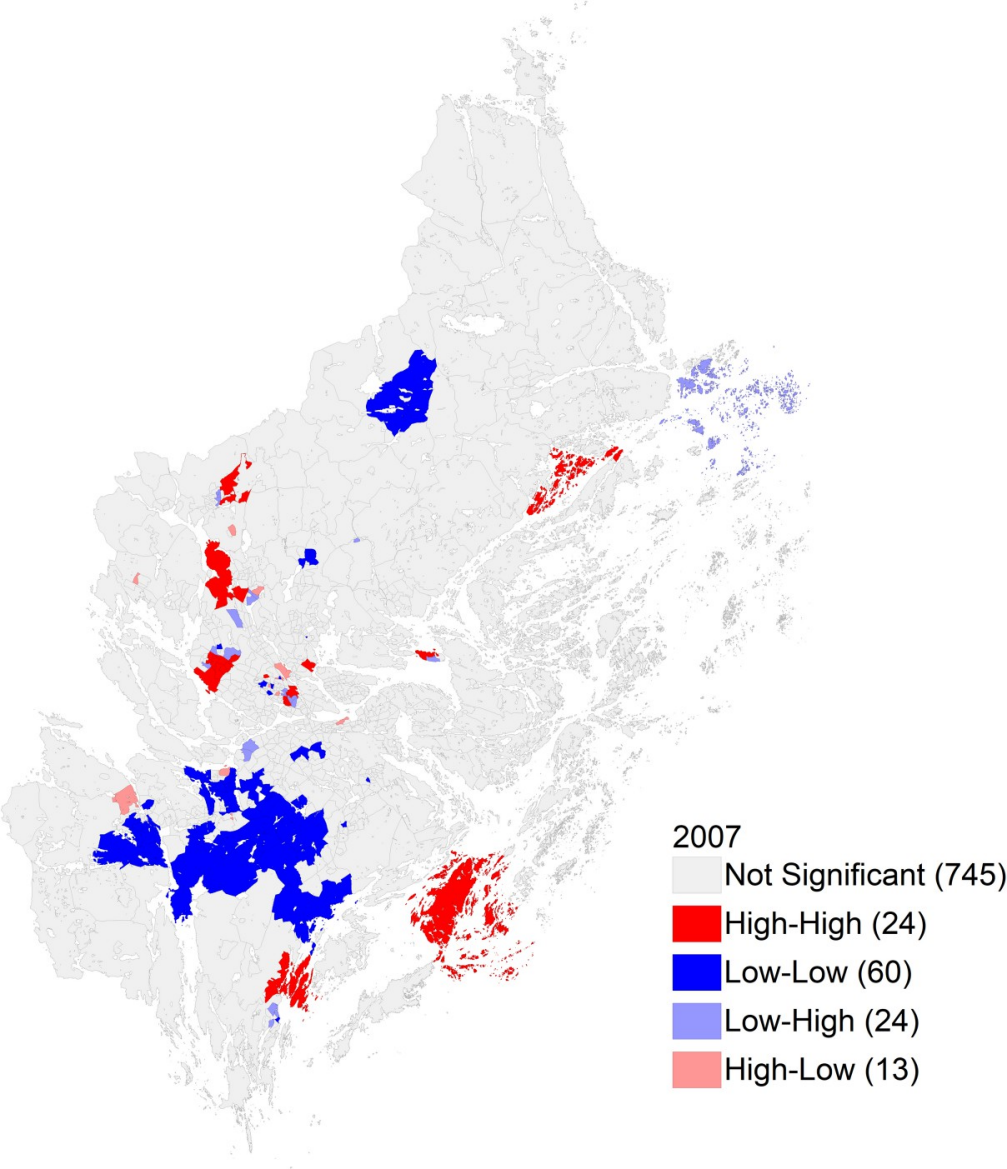
Clustering pattern of PSA testing in small areas of Stockholm for year 2007.

**Fig 6:**
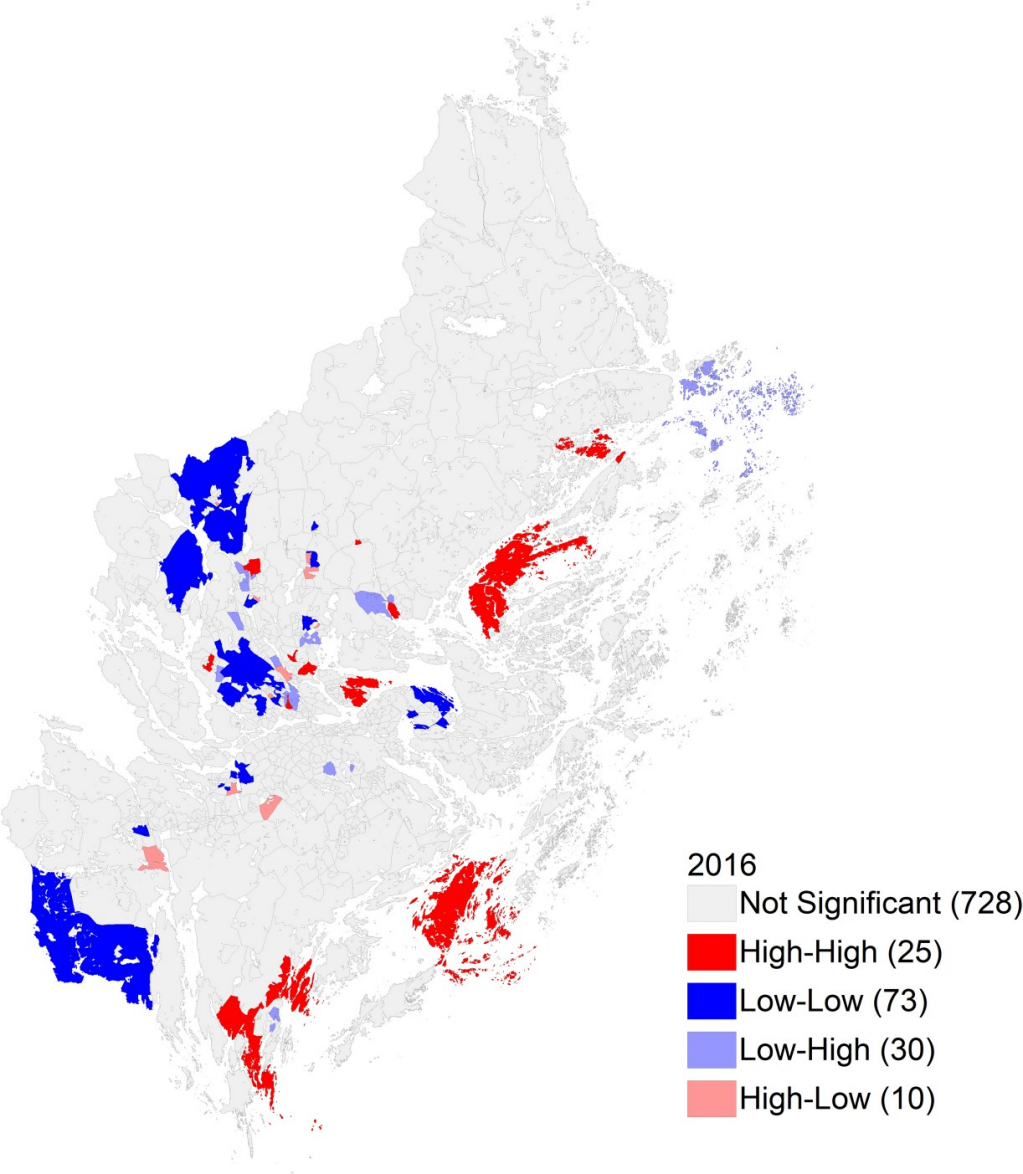
Clustering pattern of PSA testing in small areas of Stockholm for year 2016.

The small area maps showed similar trends but had marked heterogeneity within a municipality. The SAMS area in the central area, which includes metropolitan Stockholm fell in the highest category for range of proportions in 2007 (Fig 3). In particular, there was considerable heterogeneity within the central Stockholm area with smallareas falling in all the categories in 2007. For 2016, the proportion of men having a PSA test declined for most of the areas in Stockholm with no small area having a proportion higher than 25% (Fig 4). The small areas in the southern parts of Stockholm had lower proportions in 2007 compared to those in central and eastern area. From the clustering maps in Figs 5 and 6 almost all the SAMS areas with significant clustering were either in high-high or low-low clusters, with very few clusters having discordant proportions. There were significant clusters of higher proportion of men having PSA test in central Stockholm and a few scattered clusters of a lower proportion of PSA testing in southern and western Stockholm. There were more clusters of lower proportion in southern Stockholm in 2007 which shifted to western and some part of central Stockholm in 2016. There were still some high clusters in central and southern Stockholm in 2007 and 2016. The distribution of the PSA testing and the clustering pattern for the calendar years 2008–15 are described in Figs 1.1–1.8 in S1 Text and Figs 2.1–2.8 in S1 Text respectively.

## Discussion

We found widespread and consistent use of opportunistic PSA testing in the Stockholm region, with high levels of long term (5 or 10 years) testing. There was significant variation in PSA testing by small areas and municipalities, with higher levels in more affluent areas. The findings are consistent with the previous study focusing on small area variation in PSA testing [28], which found a marked variation in PSA testing in small areas of Australia. A similar study also found the variation in PSA testing by geographical areas and socio-economic position but that was restricted to men aged 50–69 years [29]. The higher proportions of testing in older age-groups in Stockholm rises concern, given frequent opportunistic testing may excerbate the risk of overdiagnosis and overtreatment [30] and low PSA values having lower probability of experiencing aggressive prostate cancer or death in these age-groups [31]. Education, income, and immigration were independently significantly associated with PSA testing in the small areas. The socio-economic variation in PSA testing could partially explain differences in prostate cancer incidence and mortality by socio-economic position. For example, increased PSA testing among affluent men has resulted in increased incidence of low-grade prostate cancers [32].

As a strength, our analysis was based on a population-based, individual-level linkage between PSA tests and men, which allows for internal migration across the study period. Moreover, men were spatially encoded for comparatively small and socio-economically homogeneous areas. As a potential limitation, we presented the proportion of men who have at least one PSA test in a year. That metric does not represent the time to the next PSA test or the number of PSA tests for men who have more than one PSA test in a year. However, modeling for the number of PSA tests for a year would be difficult due to over-dispersion between men, which would require an individual-level model with spatial random effects. The area-level regression to test the association between SEP and PSA testing could also have some limitations such as ecological fallacy, aggregation bias, and confounding hence the results should be interpreted cautiously. As a further limitation, we did not have access to more recent data after 2016. However, the PSA testing pattern for 2018–2020 may also be affected by the STHLM3-MRI study [33]. The PSA testing could also vary by general practices [34], however we did not have access to primary care data to evaluate this variation.

We found more easily interpretable spatial patterns at the municipality level, whereas there was marked heterogeneity between the SAMS areas. Possible explanations for the heterogeneity could include (a) socio-economic position at the small area level [18], (b) factors including immigration [35], (c) access to health services within the small area, and (d) the views on PSA testing between general practitioners [36]. Moreover, it is unclear how socio-economic position at the small area level may influence the PSA testing patterns in combination with individual socio-economic position.

Screening for prostate cancer using the PSA test remains controversial [37, 38]. The Stockholm region introduced an organised prostate cancer testing (OPT) pilot in 2022, where men aged 50 years are offered organised testing using PSA and MRI, with organised retesting and structured follow-up for men with raised test values. There is considerable interest to evaluate whether organised PSA testing will lead to reduced or even increased heterogeneity between areas and socio-economic factors among men who test compared with the current opportunistic testing. Over time, there have been changes in: prostate cancer diagnostics, including the introduction of MRI and reflex tests; the treatment and management of men who have been diagnosed with prostate cancer; and prostrate cancer incidence and mortality due to changes in testing, diagnostics and treatment. Future research should investigate whether PSA testing is associated with changes in these factors and outcomes.

## Supporting information

S1 Text This is supporting information text (S1 Text) which includes supplementary tables and figures. (DOCX)
