# Supplementary material for: Spatio-temporal variation in prostate cancer testing in Stockholm: A population-based study
Source: PLoS One. 2024 Aug 15;19(8):e0308254. doi: 10.1371/journal.pone.0308254 (PMC11326630; doi:10.1371/journal.pone.0308254)
Supplement: S1 Text — (DOCX) [file pone.0308254.s001.docx]

**Supplementary Tables and Figures**

|  |  | Prevalence of PSA testing in 2016 (%) | | | |
| --- | --- | --- | --- | --- | --- |
| Age group | Population | 1 year | 2 years | 5 years | 10 years |
| 40-49 | 164160 | 0.04 | 0.04 | 0.05 | 0.05 |
| 50-59 | 141629 | 0.89 | 0.98 | 1.02 | 1.04 |
| 60-69 | 106826 | 5.02 | 5.63 | 5.86 | 5.98 |
| 70-79 | 77480 | 10.43 | 11.98 | 12.75 | 13.12 |
| 80+ | 32351 | 10.68 | 12.57 | 14.03 | 14.82 |

Table 1. Prevalence of men with a PSA test following a prostate cancer diagnosis in the Stockholm region by age group and 1, 2, 5, and 10 year prevalence, 2016.


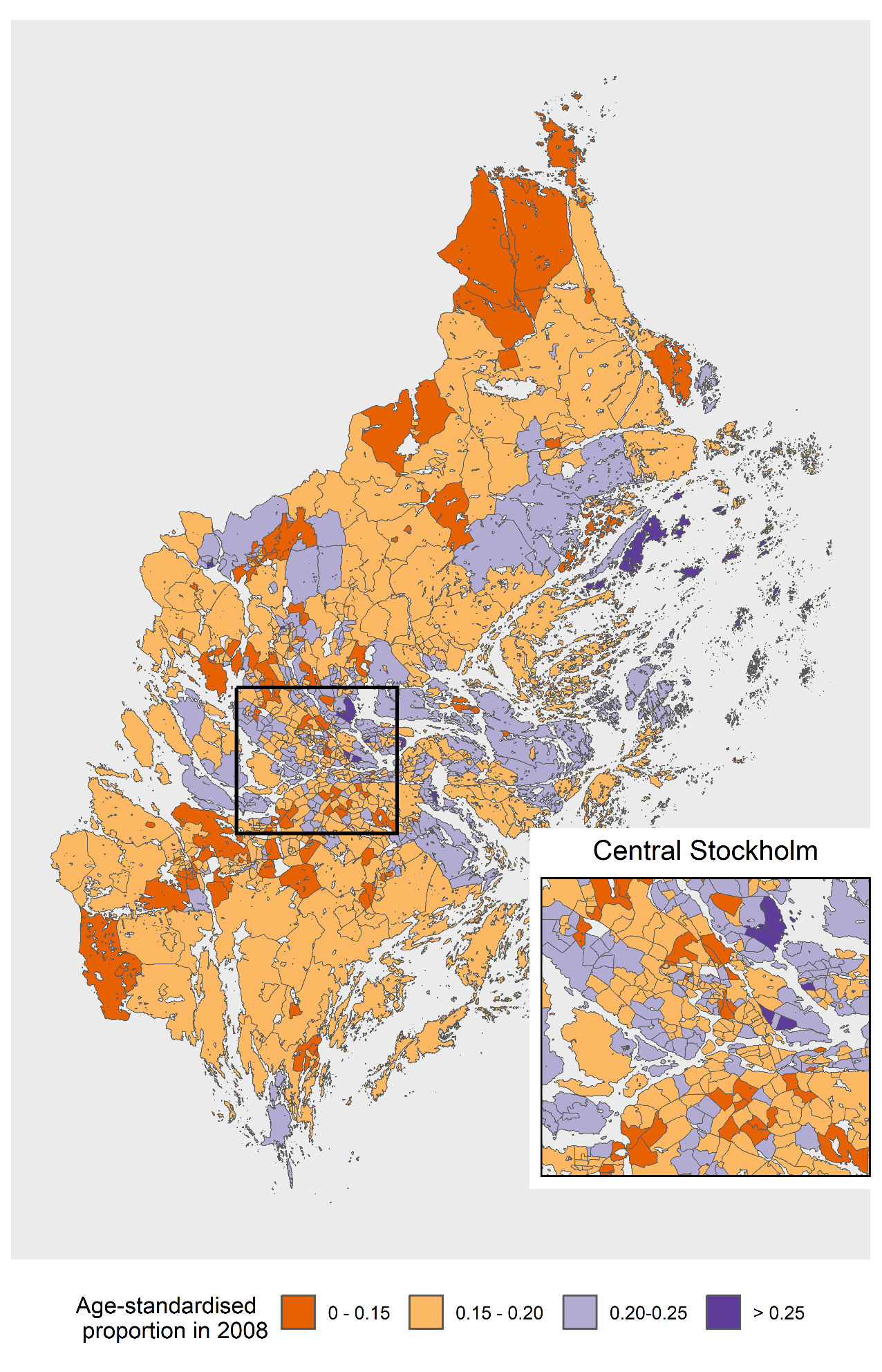


Figure 1.1) Spatial distribution for proportion of men getting a PSA test by SAMS area in the Stockholm region, 2008.


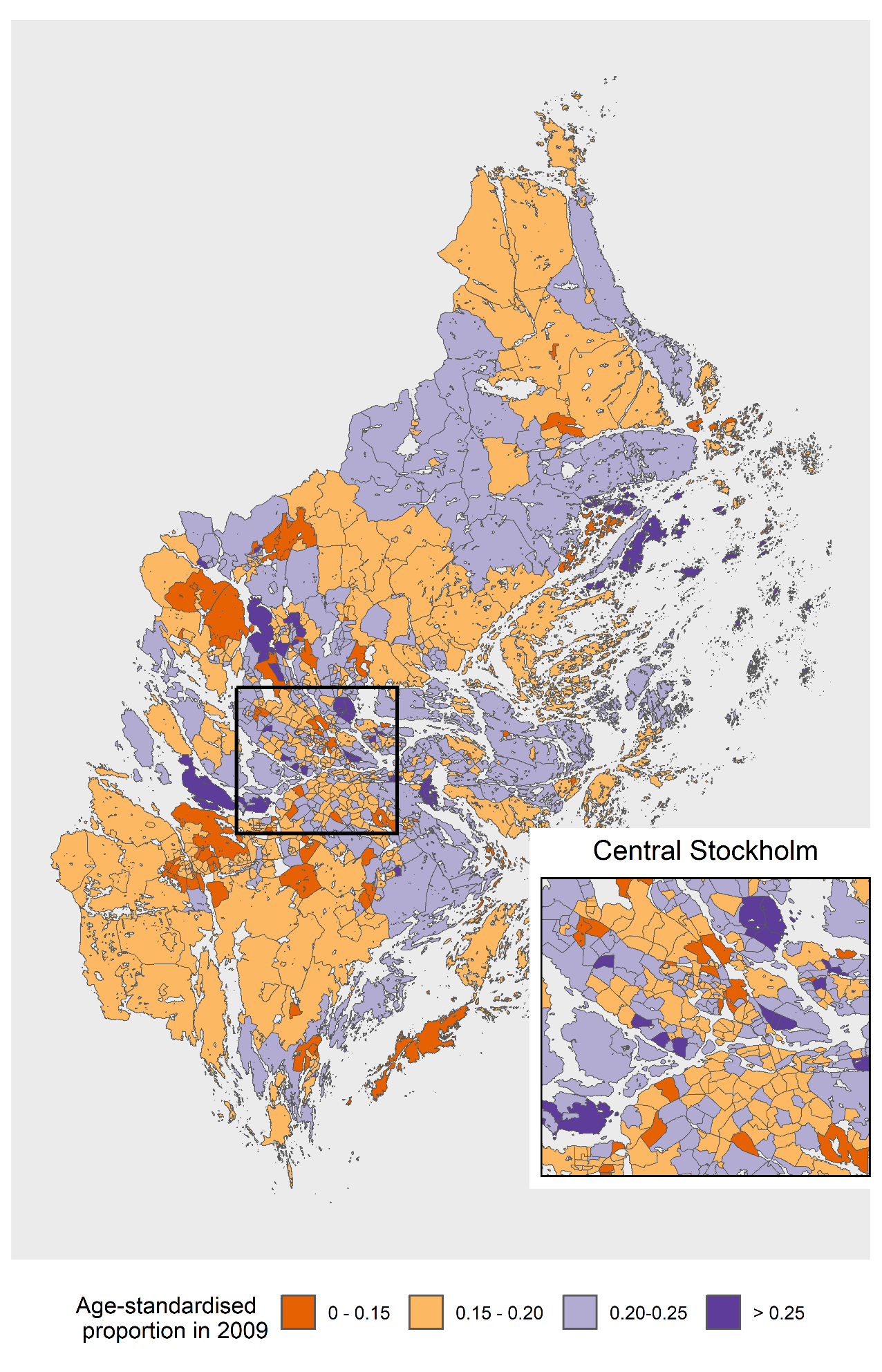


Figure 1.2) Spatial distribution for proportion of men getting a PSA test by SAMS area in the Stockholm region, 2009.


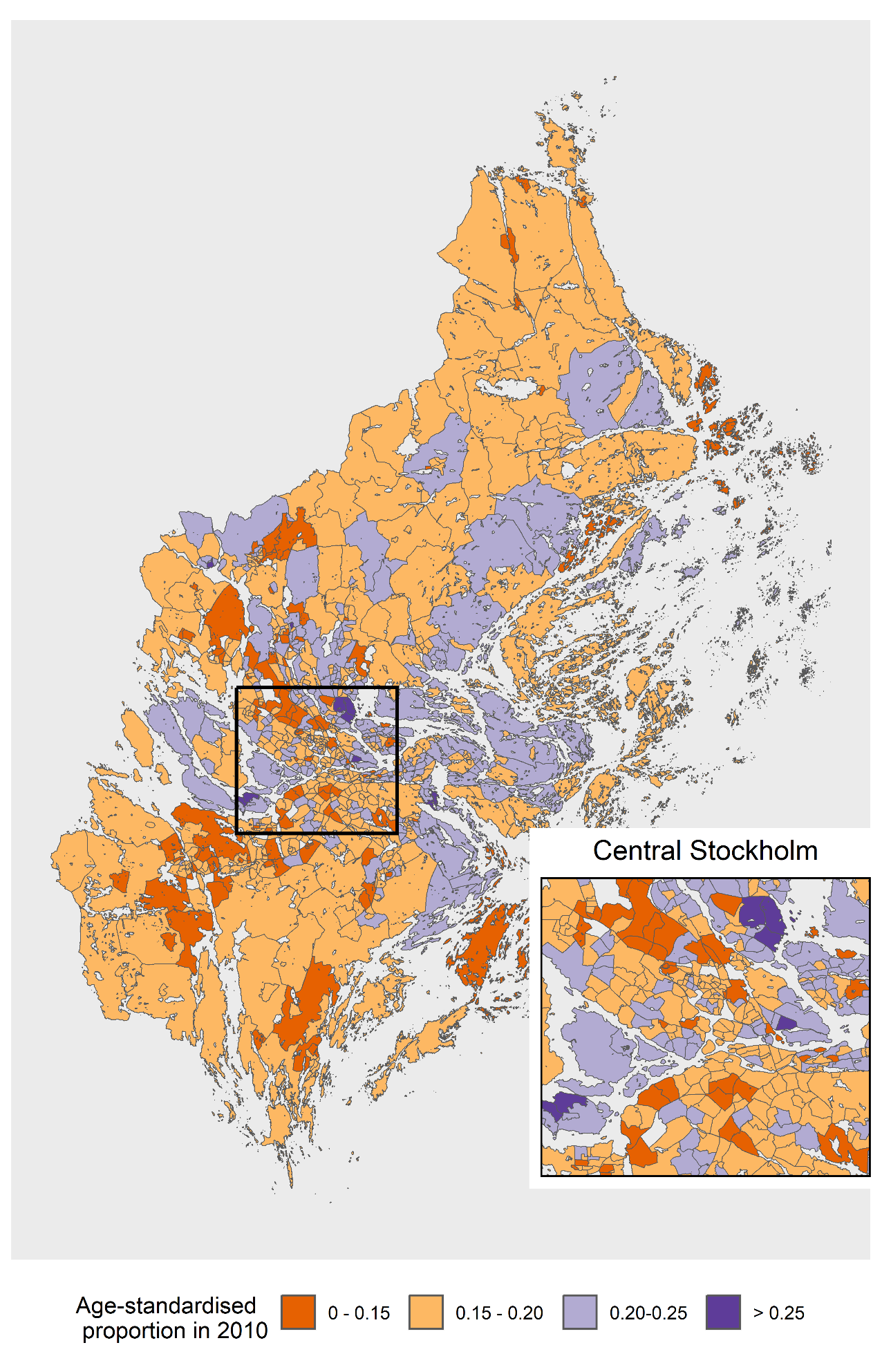


Figure 1.3) Spatial distribution for proportion of men getting a PSA test by SAMS area in the Stockholm region, 2010.


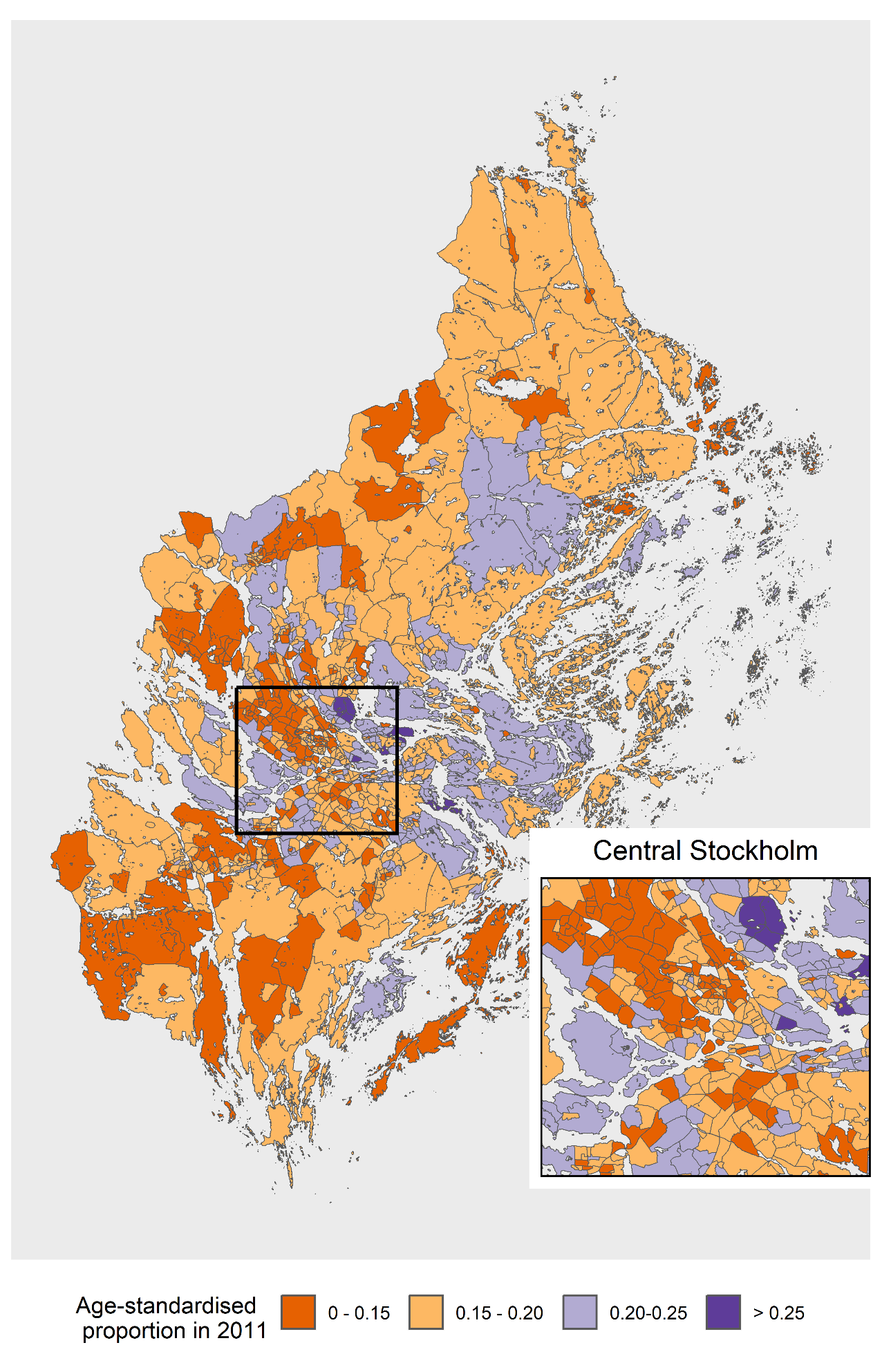


Figure 1.4) Spatial distribution for proportion of men getting a PSA test by SAMS area in the Stockholm region, 2011.


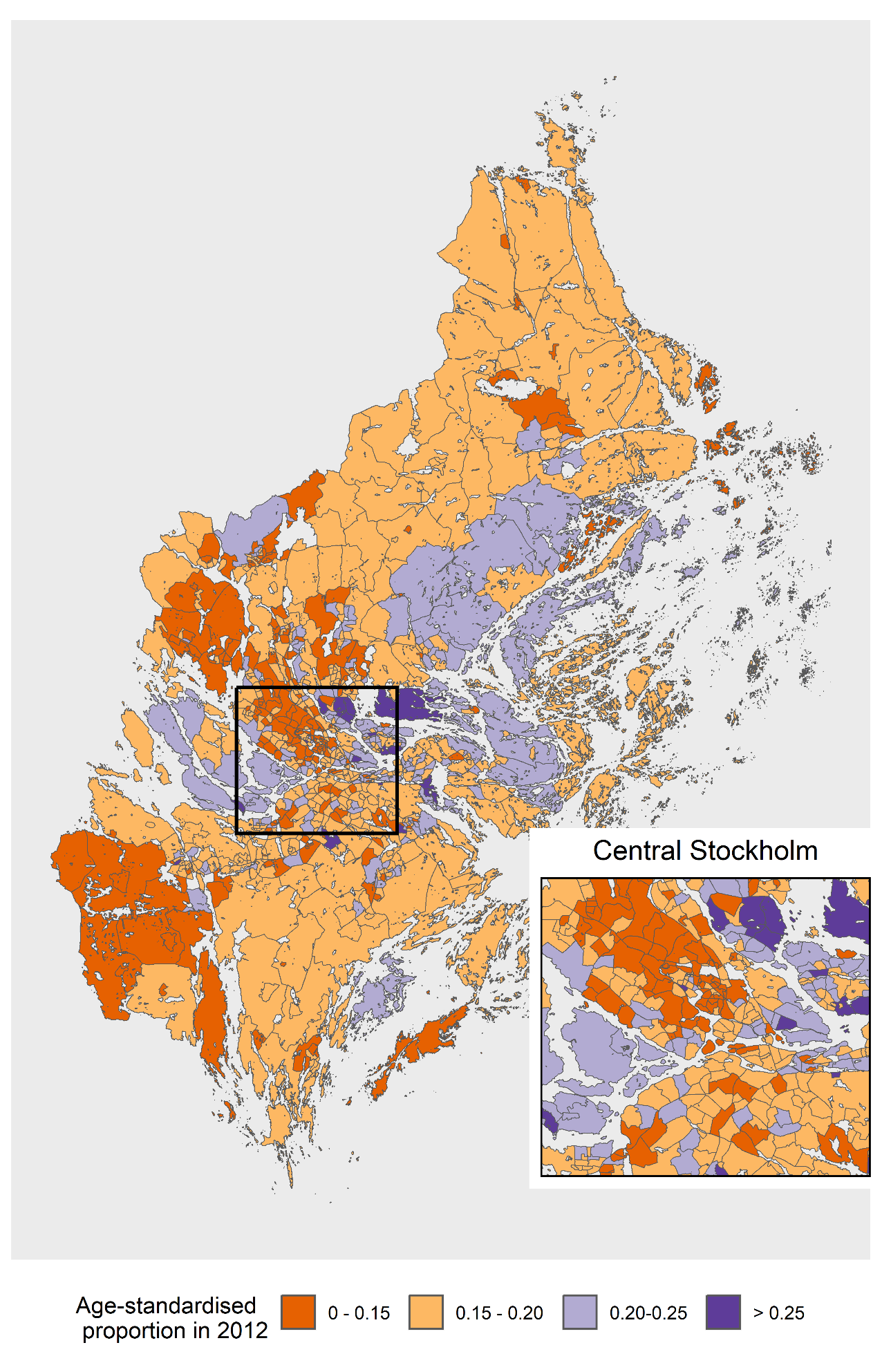


Figure 1.5) Spatial distribution for proportion of men getting a PSA test by SAMS area in the Stockholm region, 2012.


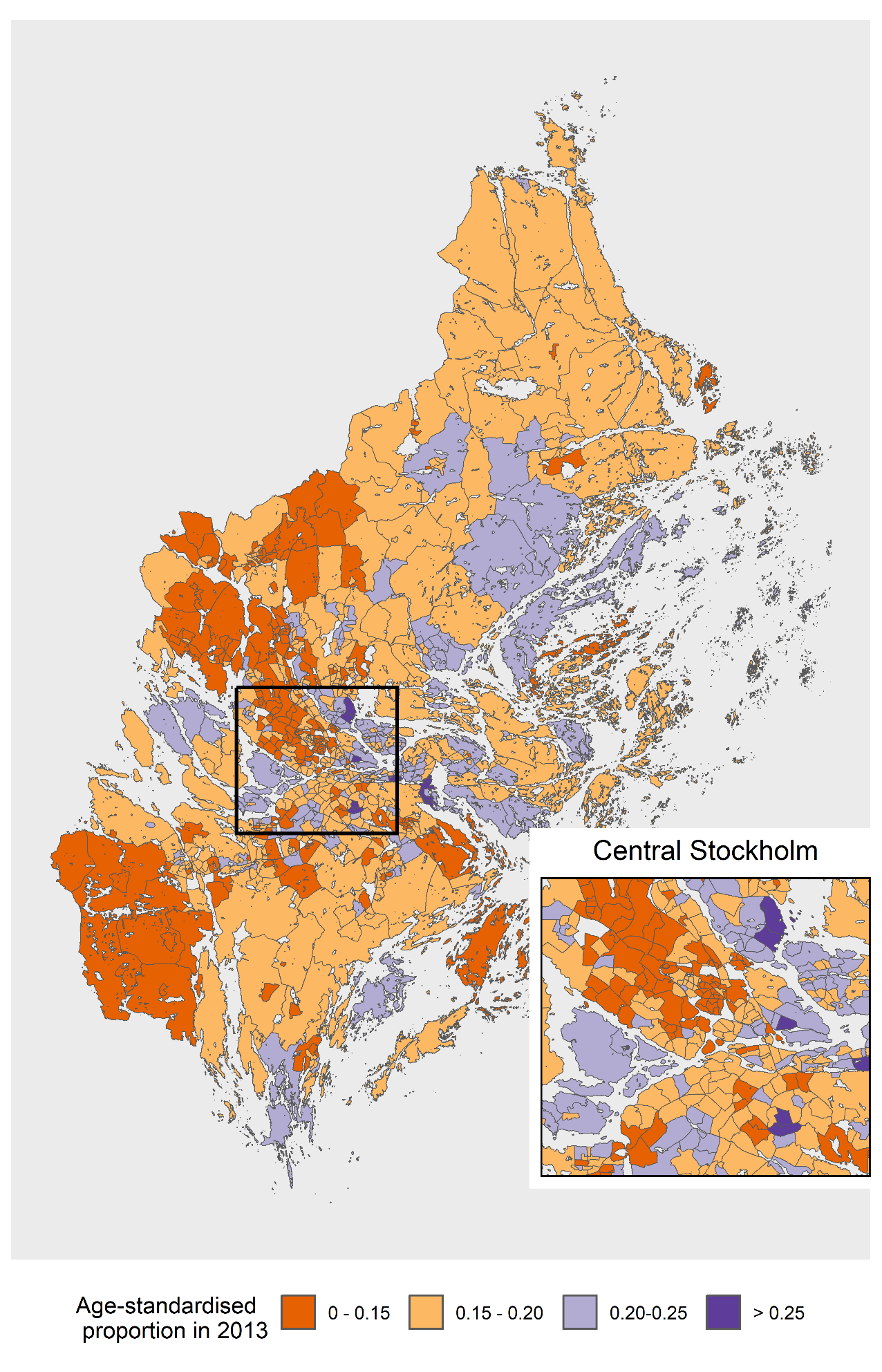


Figure 1.6) Spatial distribution for proportion of men getting a PSA test by SAMS area in the Stockholm region, 2013.


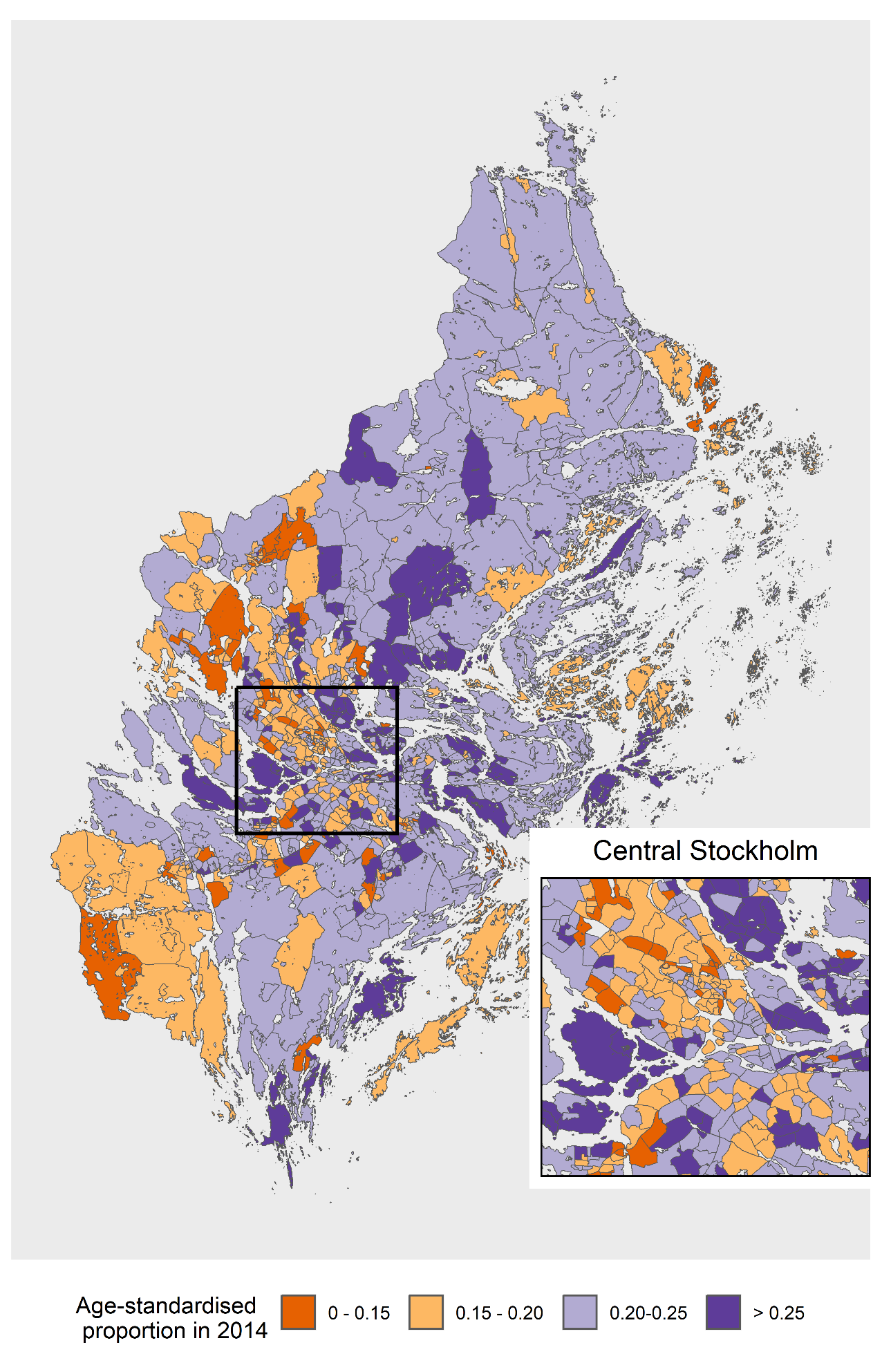


Figure 1.7) Spatial distribution for proportion of men getting a PSA test by SAMS area in the Stockholm region, 2014.


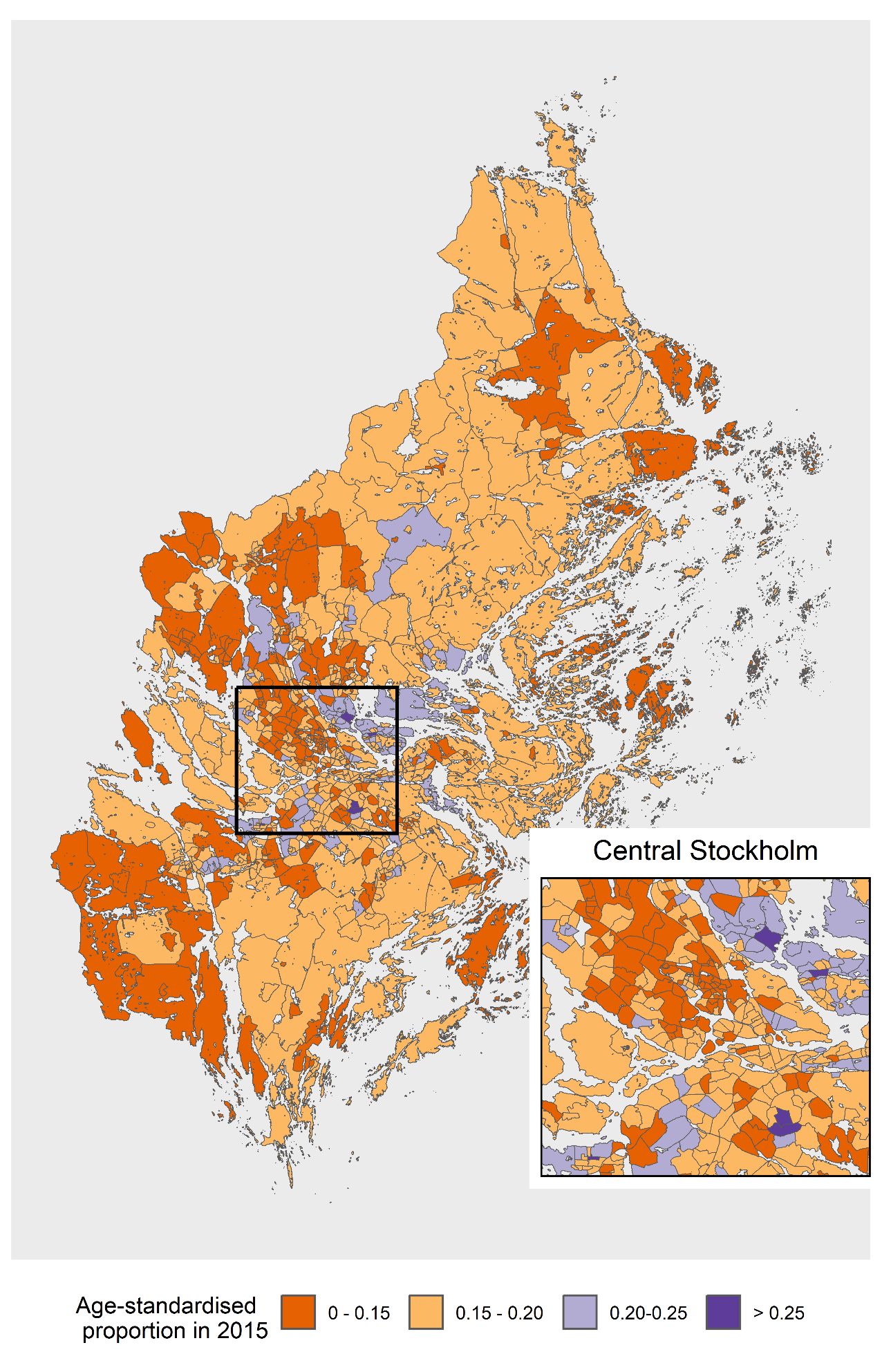


Figure 1.8) Spatial distribution for proportion of men getting a PSA test by SAMS area in the Stockholm region, 2015.


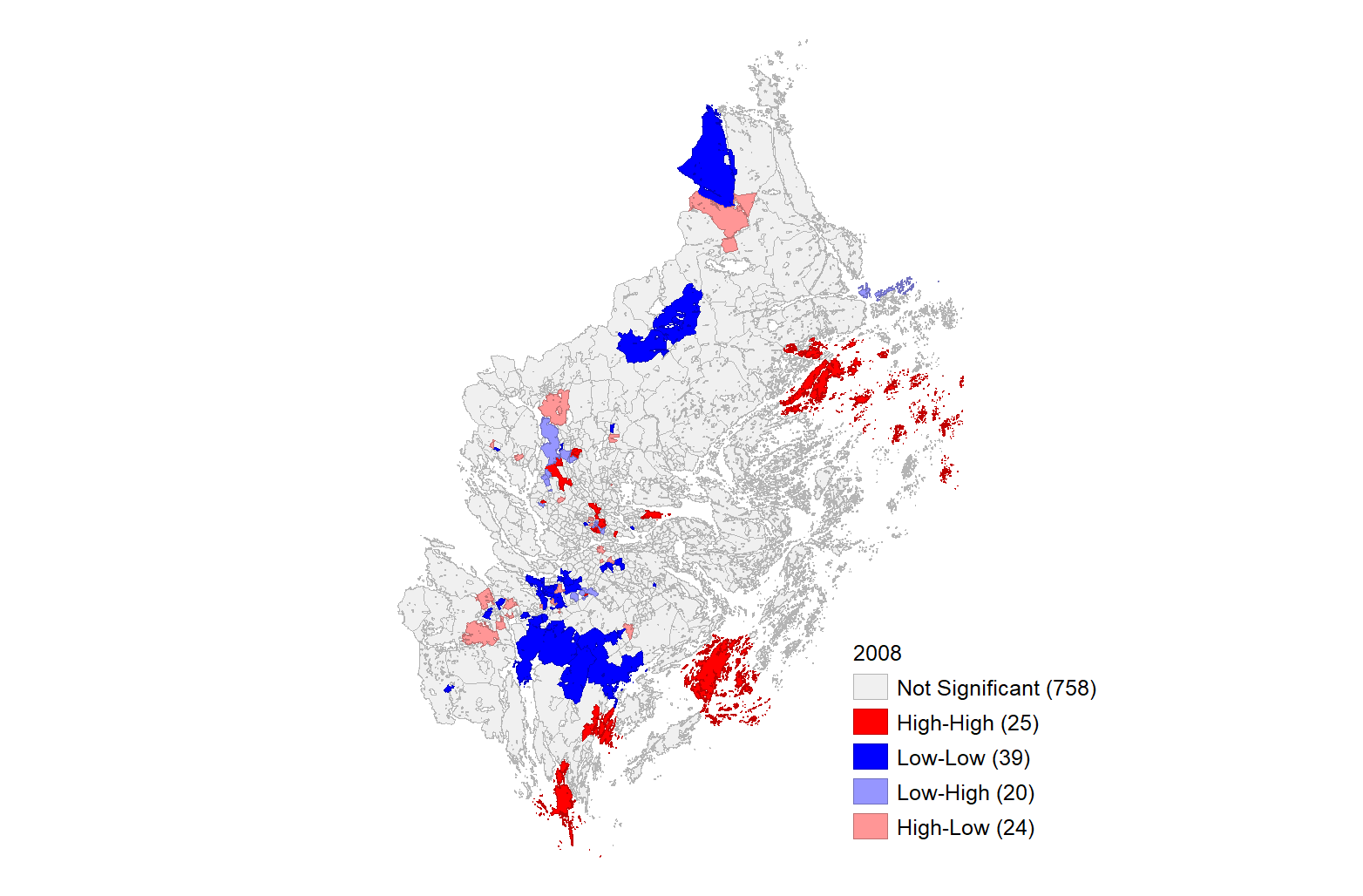


Figure 2.1) Clustering pattern for PSA testing by SAMS area in the Stockholm region, 2008.


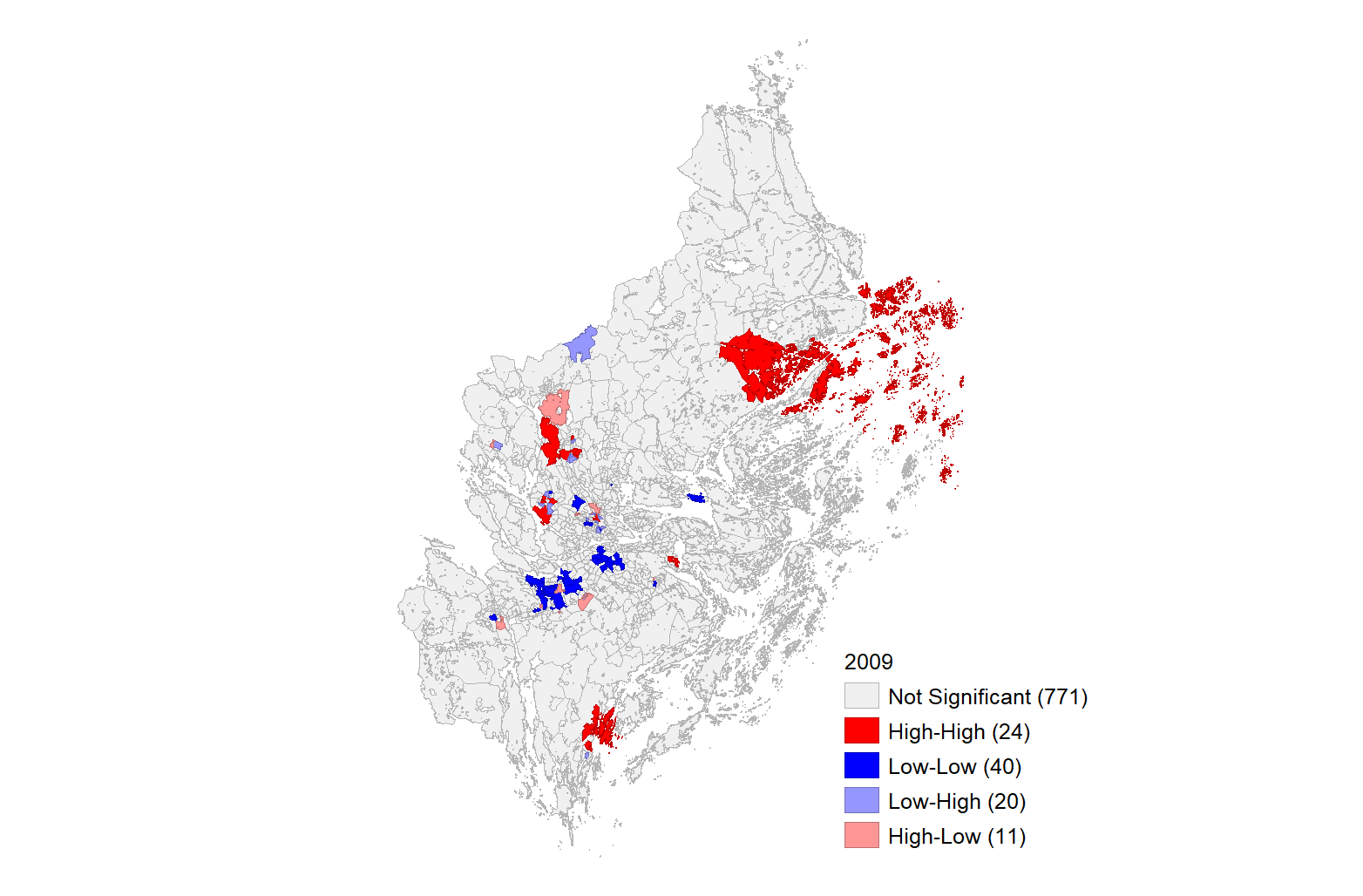


Figure 2.2) Clustering pattern for PSA testing by SAMS area in the Stockholm region, 2009.


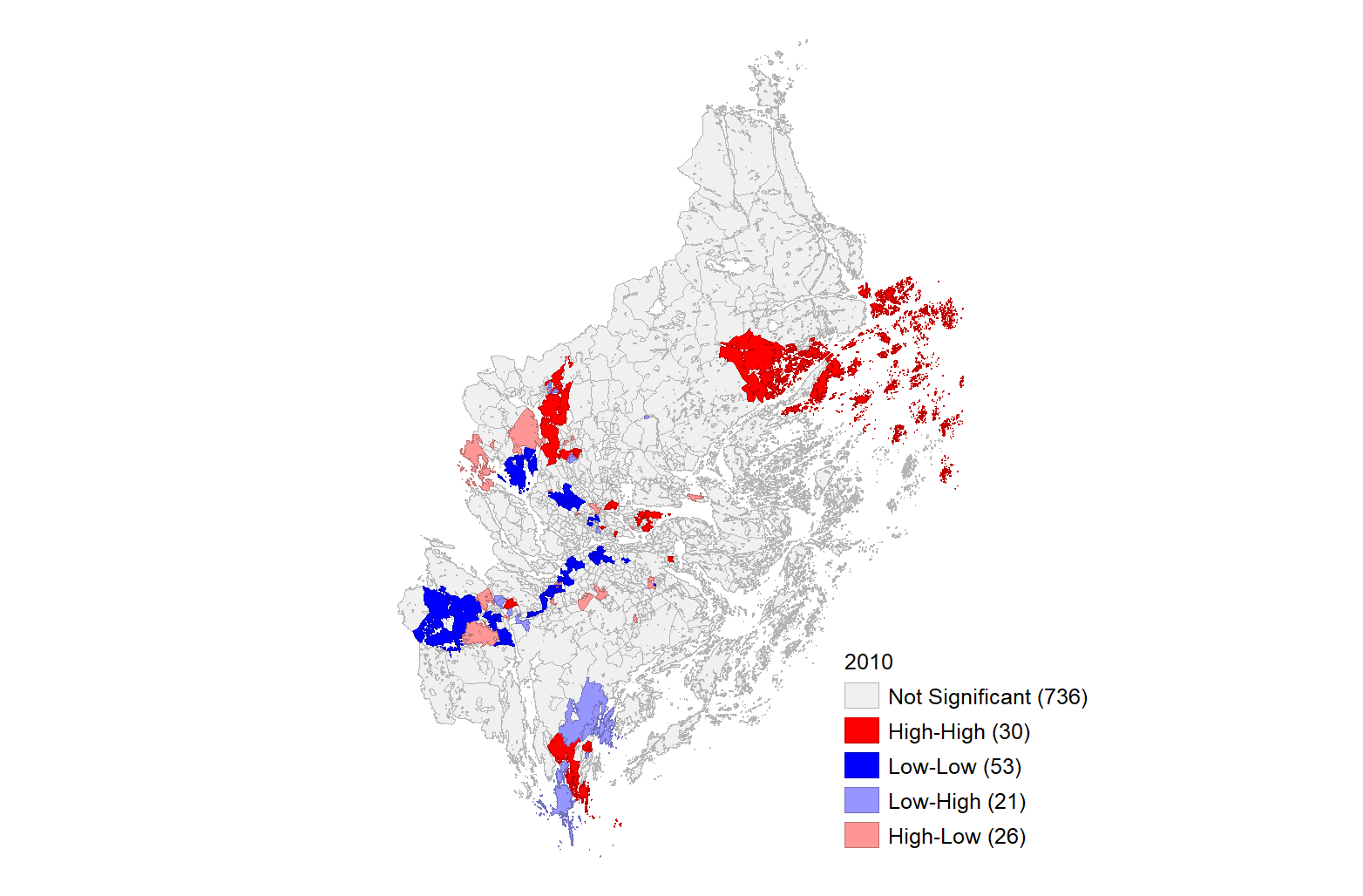


Figure 2.3) Clustering pattern for PSA testing by SAMS area in the Stockholm region, 2010.


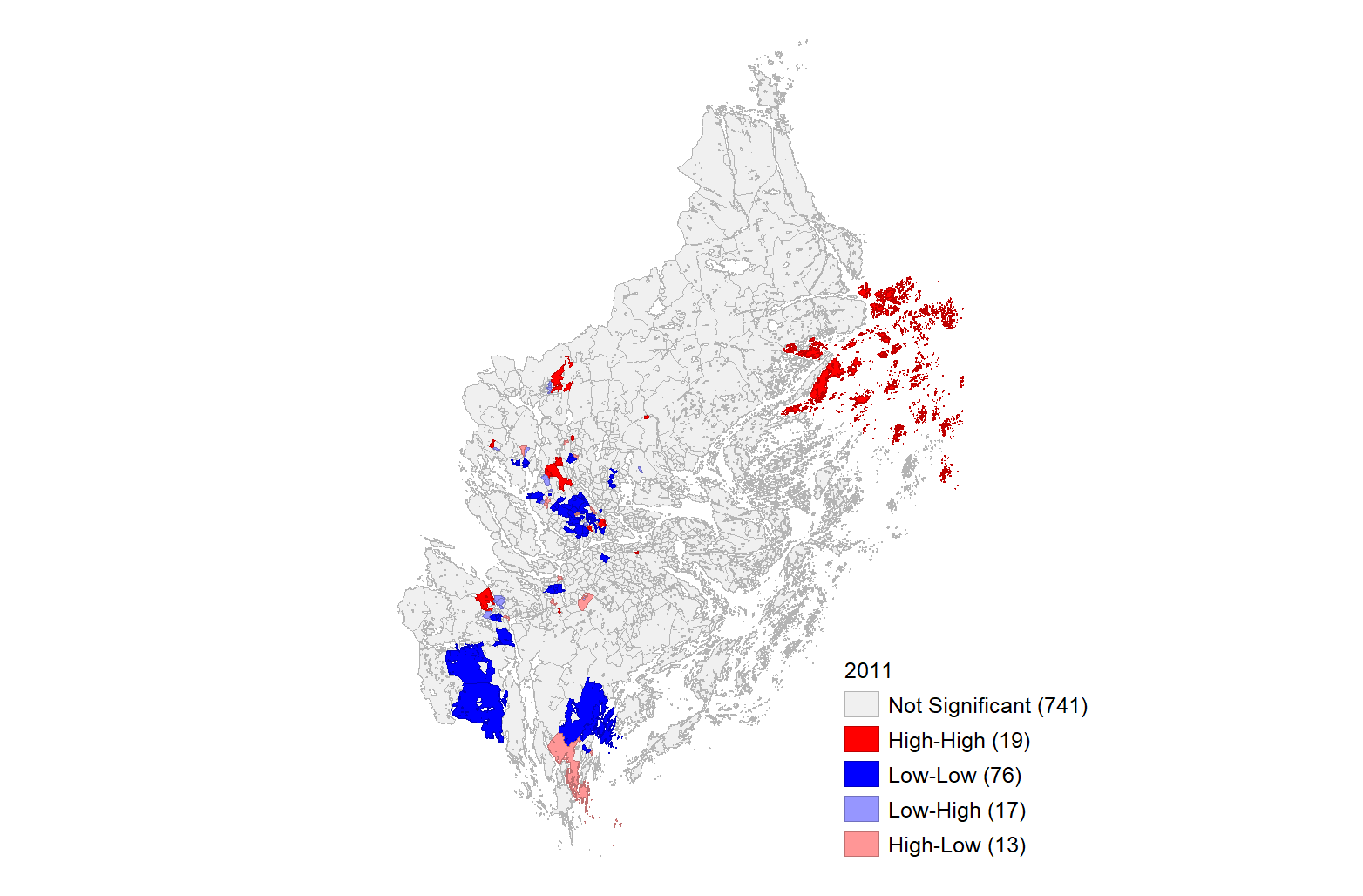


Figure 2.4) Clustering pattern for PSA testing by SAMS area in the Stockholm region, 2011.


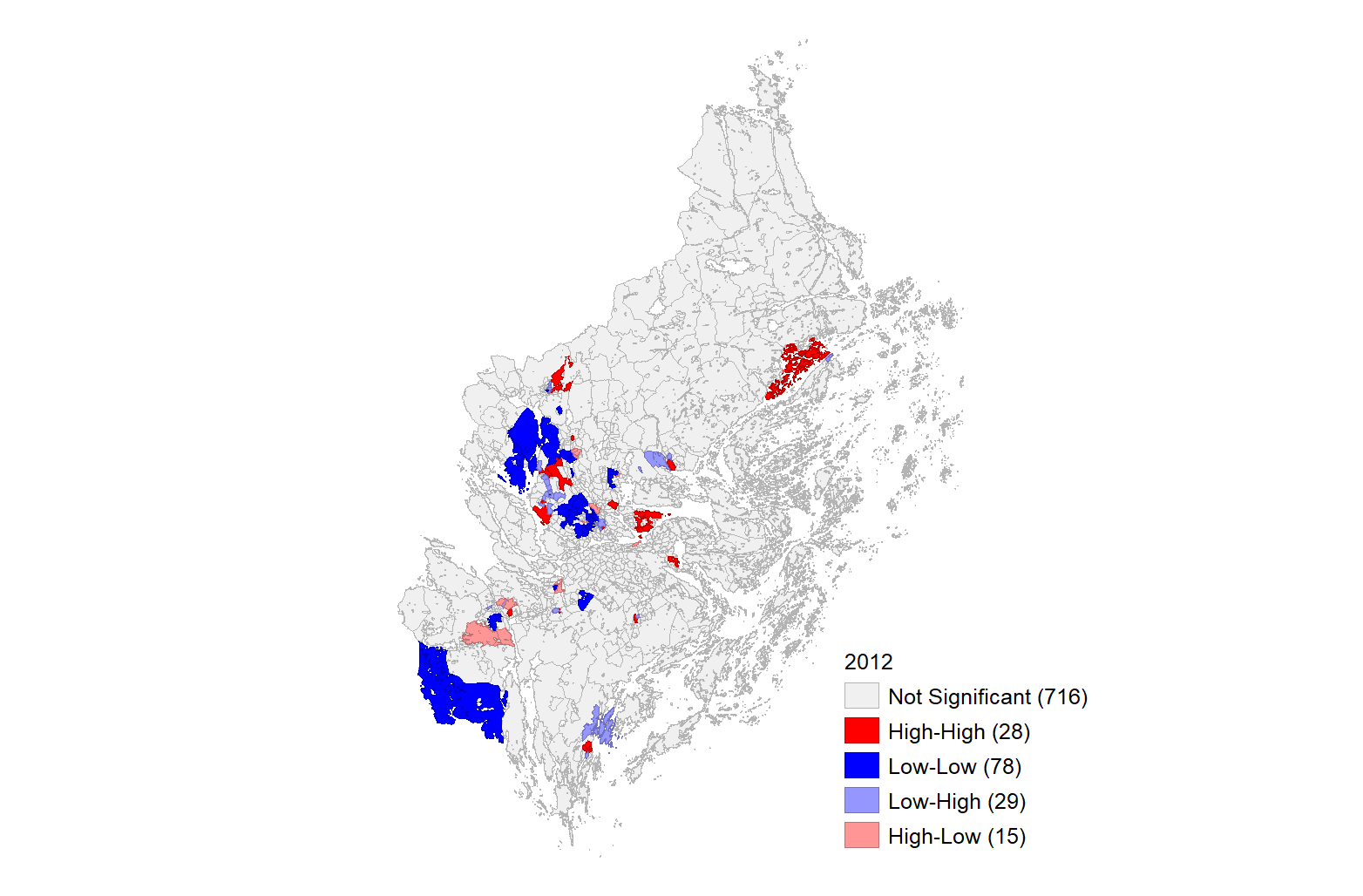


Figure 2.5) Clustering pattern for PSA testing by SAMS area in the Stockholm region, 2012.


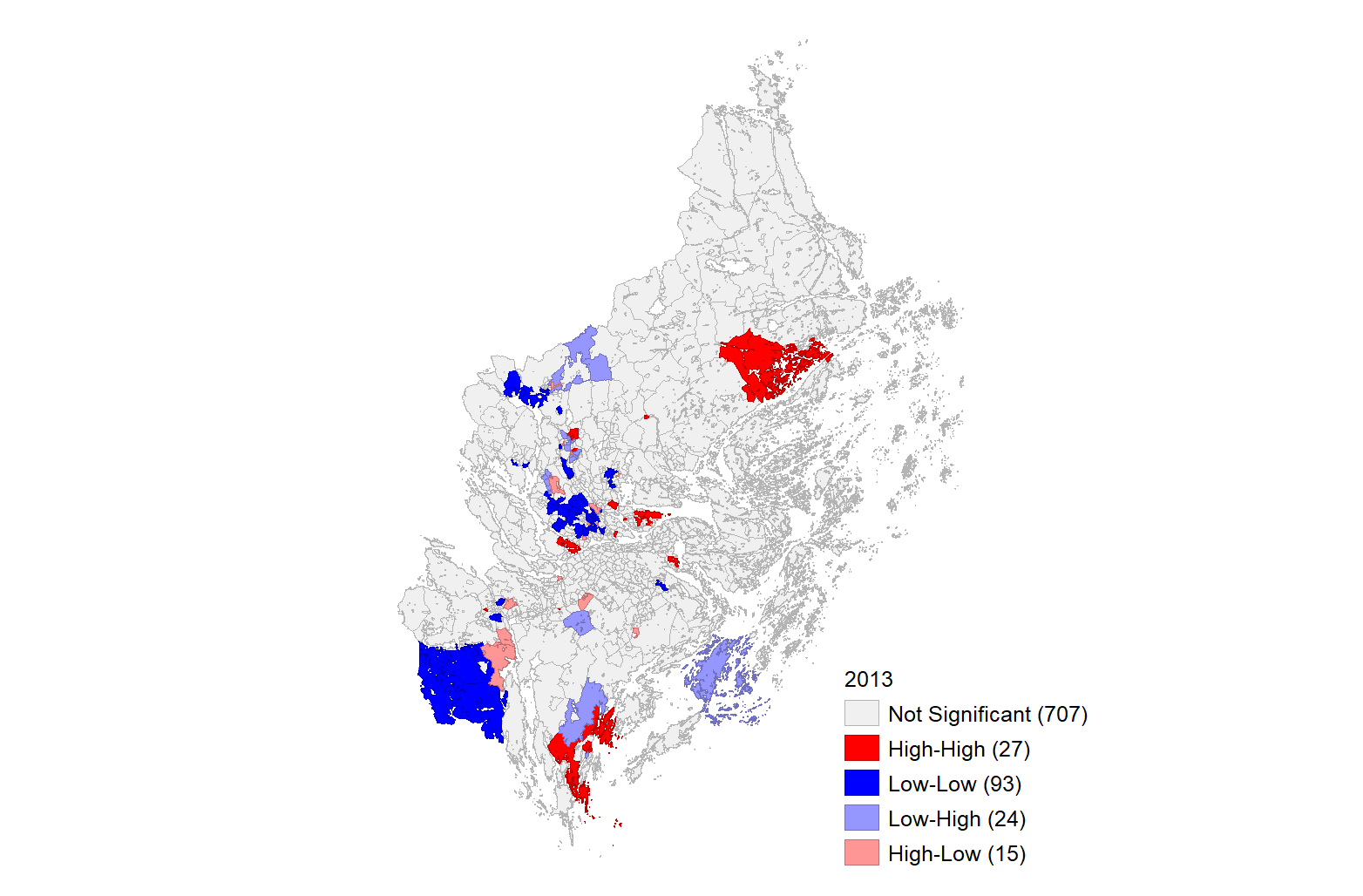


Figure 2.6) Clustering pattern for PSA testing by SAMS area in the Stockholm region, 2013.


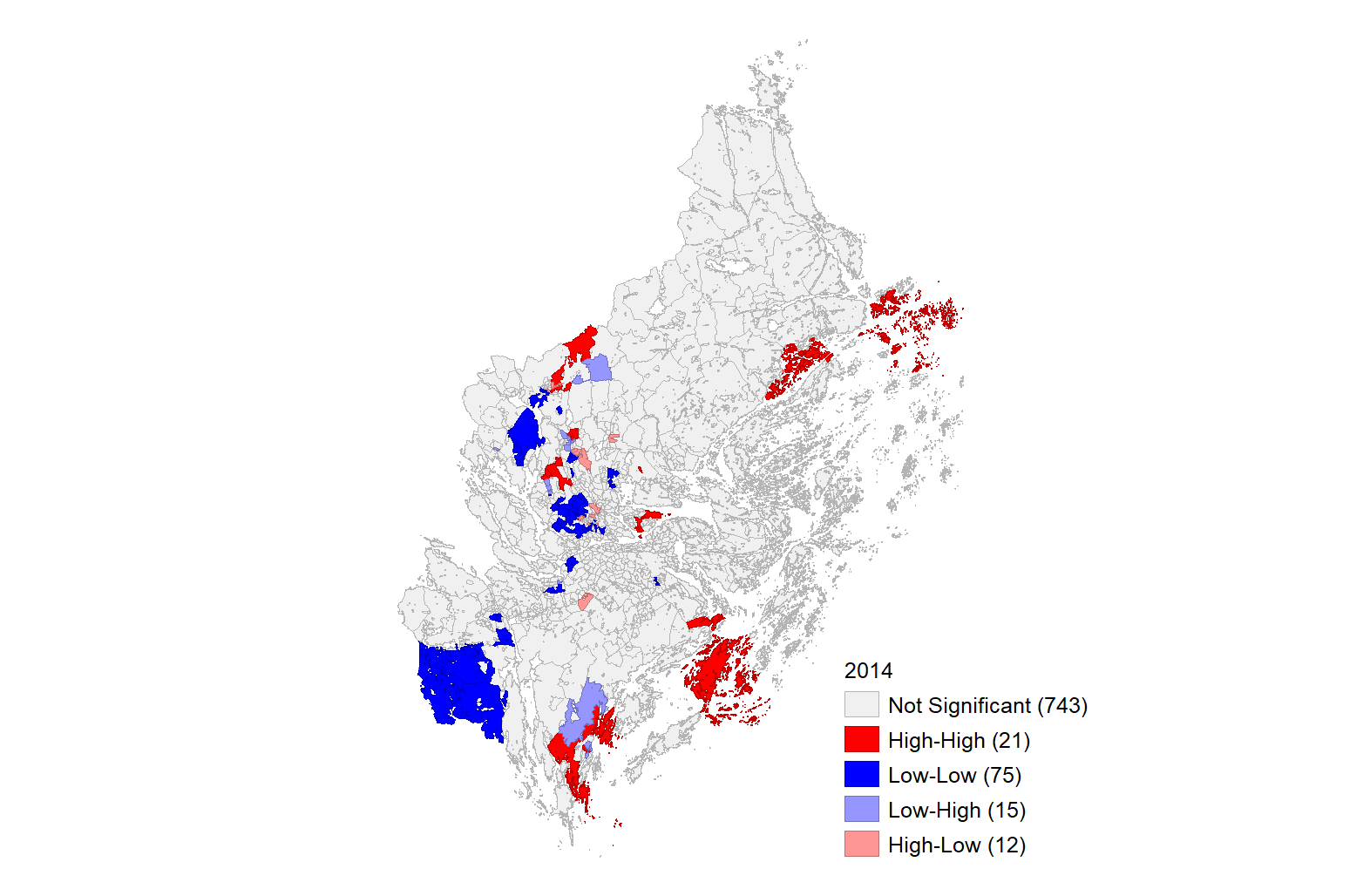


Figure 2.7) Clustering pattern for PSA testing by SAMS area in the Stockholm region, 2014.


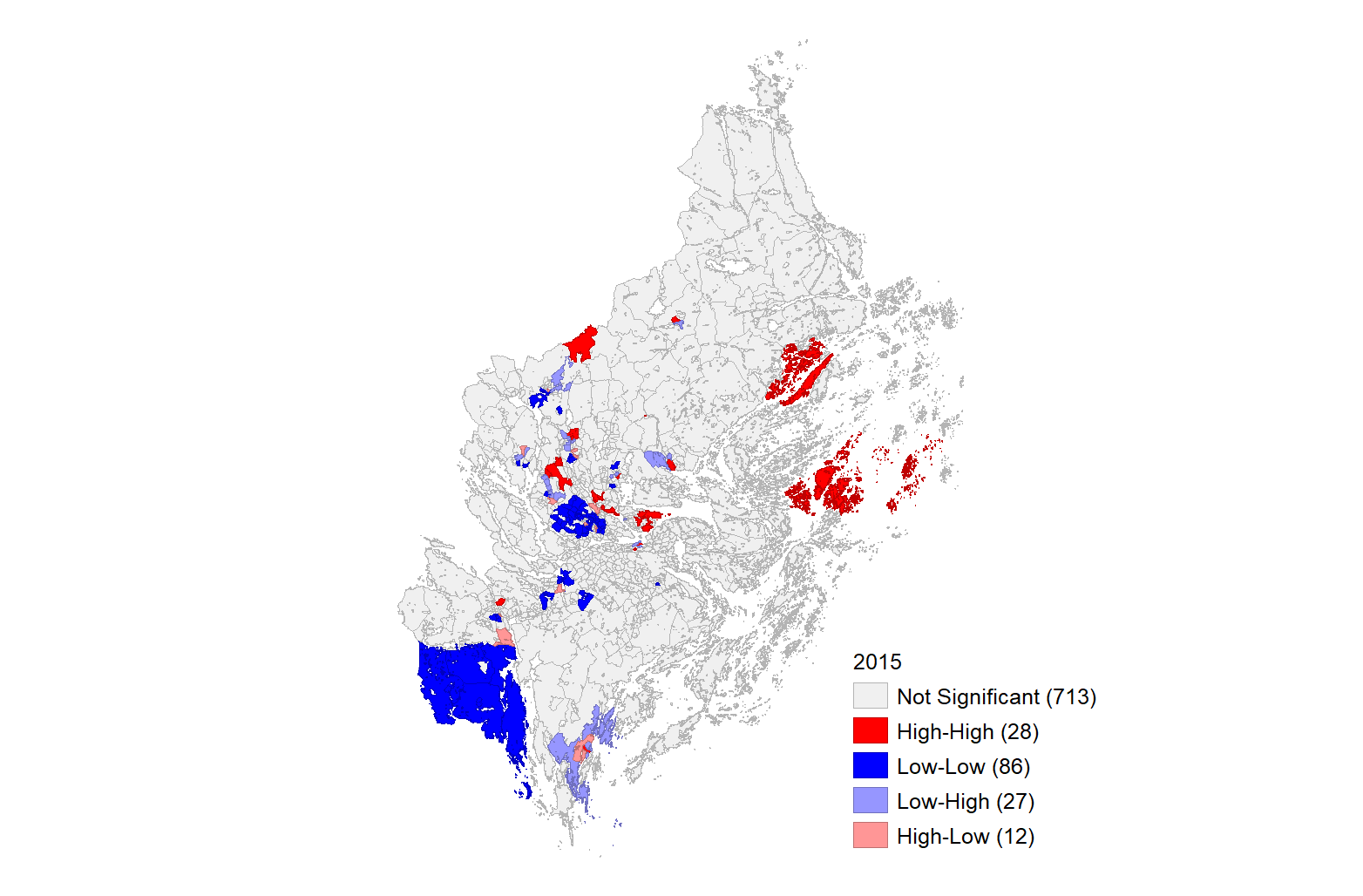


Figure 2.8) Clustering pattern for PSA testing by SAMS area in the Stockholm region, 2015.
